# Comparison of culture and real-time PCR for detection of *Bordetella pertussis* isolated from patients in Iran

**Published:** 2013-09

**Authors:** Vajiheh Sadat Nikbin, Fereshteh Shahcheraghi, Masoumeh Nakhost Lotfi, Seyyed Mohsen Zahraei, Masoumeh Parzadeh

**Affiliations:** 1 Pertussis Reference Laboratory, Department of Bacteriology, Pasteur Institute of Iran, Tehran; 2 Center for Disease Control, Ministry of Health and Medical Education, Tehran, Iran

**Keywords:** *Bordetella pertussis*, real-time PCR, IS*481*, BP283

## Abstract

**Background and Objective:**

Due to contagiousness of pertussis, a rapid and sensitive method for diagnosis is required to initiate the treatment and interrupt its transmission.

**Materials and Methods:**

To detect *B. pertussis* strains, we used two real-time PCR targeting IS*481* and BP283 sequences and compared factors influencing culture and real-time PCR results.

**Results:**

Totally, 779 specimens were collected from patients among which 11 (1.4%) were culture positive. Using IS*481* and BP283 primers, 122 (15.6%) and 100 (12.8%) were diagnosed as infected specimens respectively. There were significant relationships between the real-time PCR method for diagnosis of *B. pertussis* and age, sex and vaccination of patients before sampling.

**Conclusion:**

The real-time PCR is superior and much more sensitive than culture for diagnosis of *B. pertussis*. However, the sensitivity was improved when both IS*481* and BP283 were used. Correct sampling and transportation of specimen also improved the detection rate in our research.

## INTRODUCTION

Whooping cough (pertussis) caused by *Bordetella pertussis* is a hazardous respiratory illness mostly among infants (1–3). Despite decreasing the incidence of this disease by extensive vaccination around the world, pertussis has been re-emerged especially in those under 5 months old and also over 10 years in the last decade (2, 4–9). This may be due to several factors including adaptation of vaccine-induced immunity among the *B. pertussis* strains (strain polymorphism), waning vaccine induced immunity, incomplete protection from vaccination and expansion of strains that are antigenetically distinct from vaccine strains (5, 10–14). Moreover, changes in case definition and improvement in diagnosis and reporting may result in higher incidences of pertussis infections (2, 12).

Due to the importance of pertussis as a contagious disease, it is necessary to utilize a rapid and sensitive detection of *B. pertussis* to interrupt its transmission (15–18). Real-time PCR, using insertion sequence IS*481*, is the most common molecular method for the rapid detection of *B. pertussis* (4, 16, 19–21). The genome of *B. pertussis* contains high copy number of this insertion element (22), however, IS*481* element has also been found in *B. holmessi* and some strains of *B. bronchiseptica* (23, 24)

Therefore, this target may make some false positive results and is not very specific target in laboratory diagnosis of *B. pertussis*. However, lack of specificity of this target has led the researches to alter other sequences such as pertactin gene (25), promoter of pertussis toxin gene (*ptx-*Pr) (21, 26, 27), porine gene (28, 29), single copy PCR target BP3385 (30), BP283 and BP485 (31), as a means of confirming IS*481* for the detection of *B. pertussis* in clinical samples.

As these sequences have only one copy in *B. pertussis* genome, these target sequences can be used in combination with IS*481* to improve the accuracy of *B. pertussis* detection for laboratory diagnosis (31).

There is no sufficient information about incidence of *B. pertussis* infection in our country. In this study we used IS*481* and BP283 targets in real-time PCR for detection of clinical *B. pertussis* strains isolated from patients and compared factors that influence culture and real-time PCR for detection of this bacterium in clinical samples.

## MATERIALS AND METHODS

### Specimen collection

A total of 779 specimens (two dacron-tipped swabs per specimens) were collected from pertussis suspected patients and transported in Regan-Lowe transport medium to the Pertussis Reference Laboratory at the Pasteur Institute of Iran during May 2009 to December 2010. One swab was cultured and streaked on to fresh Bordet Gengou medium and Regan-Lowe medium (provided from Difco Laboratories) containing 10% defibrinated horse blood with and without cephalexin (40µg/ml) (Sigma Chemical Co., USA). After sufficient incubation of the isolates at 35°C for up to 10 days in a humid atmosphere, suspected Gram negative coccobacilli, non motile, catalase and oxidase positive colonies selected for further confirmation tests. Then API 20E System used for biochemical test and specific slide agglutination reaction with *B. pertussis* antiserum performed to confirm *B. pertussis* strains (Difco Laboratories) (32, 33).

### DNA extraction

The other swab was subjected for DNA extraction of the isolates in order to obtain DNA templates for real-time PCR. Nucleic acid of the isolates was extracted using high pure PCR template purification kit according to the manufacturer instruction (Roche Applied Science).

### Real-time PCR

Taqman PCR assay was performed based on IS*481* target and confirmed by BP283 target [GeneBank accession number BX470248] (16, 31), in Applied Biosystems 7500 instrument using the thermal program of 15 min at 95°C followed by 50 cycles of 30s at 95°C, 30s at 55°C. In order to confirm PCR performance without any inhibitors in master mix amplification of the human GAPDH gene in each run used for internal control (IC). The PCR was performed in a total volume of 30 µl containing 1X master mix (Roche Applied Science), 7.5 µM of each primers and probe (Table 1) and 5µl of extracted DNA that finalize PCR combination.

**Table 1:** Primer and probe sequences used in real-time.

PCR target	Forward primer (5'–3')	Reverse primer (5'–3')	Probe (5'–3')
***IS481***	ATCAAGCACCGCTTTACCC	CACACCTACCAGAACTCCCAA	FAM-AATGGCAAGCCGAACGCTTCA-TAMRA
**Bp283**	CAGGCACAGCACGTATTGCG	GACGATTACCAGCGAGATTACGA	FAM-CCGCCATCGCAACCGTCGCATTCA-TAMRA
**IC**	CCACTCCTCCACCTTTGA C	ACCCTGTTGCTGTAGCCA	VIC-TTGCCCTCAACGACCACTTTGTC-TAMRA

We also examined the specificity of target BP283 using *Bordetella parapertussis* ATCC 15311 and non-*Bordetella* strains such as *E. coli* ATCC 35218, *Pseudomonas aeruginosa* ATCC 27853, *Klebsiella pneumoniae* ATCC 9997, *Streptococcus pneumoniae* ATCC 49619, *Staphylococcus aureus* ATCC 6538, *Staphylococcus epidermidis* ATCC 12228, *Haemophilus influenzae* ATCC 10211, *Streptococcus pyogenes* ATCC 19615 and *Streptococcus agalactiae* ATCC 12386.

### Statistical analysis

Statistical analysis of some variables for detection of *B. pertussis* by real-time PCR (using BP283 target) including age (grouped as ≤2, 2–10, ≥ 10 years), gender, antibiotic treatment of patients, vaccination and cough symptoms in patients was performed via the Chi**-**square test.

## RESULTS

In this research 11out of 779 specimens (1.4%) were culture positive and 122 (15.6%) and 100 (12.8%) were diagnosed as infected using real-time PCR with IS*481* and BP283 target primers, respectively.

All strains of *B. pertussis* recovered by culture were also positive by real-time PCR using both IS*481* and BP283 (sensitivity 100%) (Table 2). Nine isolates of culture positive (81%) and 79 PCR positive specimens using BP283 (89%) were isolated from patients with 2 years old or younger. The results for *B. parapertussis* and other species of bacteria were negative using BP283 target.

**Table 2:** Sensitivity of Real-Time PCR assay using primers IS*481* and BP283 compare to culture.

Assay	Positives No (%)	Sensitivity%
**Culture**	11 (1.4)	Not applicable
**PCR (IS** ***481*** **)**	122 (15.6)	100
**PCR (BP283)**	100 (12.8)	100

We have several type of errors in sample collection including using cotton swabs instead of dacron swabs, antibiotic treatment of patients before sampling, sending the samples in time longer than 72 hours to laboratory, specimens delivered in unsuitable or expired transport medium (data not shown) (3, 32). Although, dacron and rayon swabs are an excellent choice for both PCR and culture for detection of *B. pertussis*, one of 11 isolates of *B. pertussis* that recovered in culture in this research was from a sample that was collected by use of cotton swab.

The results of statistical analysis showed that there were significant relationships between the results obtained from real-time PCR method and age, sex, and vaccination of patients before sampling (P < 0.05). Such relationship was not observed when culture was used.

## DISCUSSION

In spite of high specificity of culture for diagnosis of *B. pertussis*, low sensitivity of this method has led the researcher to use molecular method. The positive results based on IS*481* sequence (a multiple copy target) in PCR might be regarded as evidence for the infection caused by *Bordetella* spp., however, IS*481* was found in both *B. pertussis* and *B. holmesii* genome (16, 31). This target has also been identified in some strains of *B. bronchiseptica* (23, 34). However, *B. holmesii* has infrequently been isolated from nasopharyngeal samples (35, 36). Therefore, to ensure high specificity, use of more than one target in PCR assay has been suggested (37, 38).

Previous studies have shown that promoter of pertussis toxin and a porin gene were specific in *B. pertussis* (28, 29, 39). In 2008, Probert *et al* also introduced two BP283 and BP485 genes as being specific for *B. pertussis* (31). Out of total pertussis suspected specimens sent to the Pertussis Reference Laboratory of Pasteur Institute of Iran (n = 779) during thirty months of study, the rate of *B. pertussis* diagnosed by culture was 1.4% (Table 2). However, the rate of Real Time PCR positive cases in our research was 12.8% (based on BP283 target). Other studies in Germany and Denmark indicated similar rate of 13% and 15%, respectively (15, 16).

Like previous studies our results showed that real-time PCR is superior and much more sensitive than culture for diagnosis of *B. pertussis* (Table 2) (28, 37, 40). The number of cases diagnosed by using BP283 target (n = 100) in this study was almost nine times higher than those diagnosed by culture (n = 11). However, difference between culture and PCR positive strains in our study is higher than other investigations (2, 15, 21, 31). The probable reasons for this difference might be due to the time and techniques of sampling and delay between sampling and laboratory testing (15).

Our study showed that out of 122 total PCR positive samples examined by IS*481* target, 100 samples were positive using confirmative primers BP283 as well. Serial 10-fold dilutions of *B. pertussis* suspension were used to determine the limit of detection for each assay. Following nucleic acid extraction and real-time PCR, the limits of detection for the IS*481* and BP283 assays were 1.0, 0.1 CFU/reaction, respectively. Multiple copies of the IS*481* target sequence in the *B. pertussis* genome cause lower detection limit compared to BP283. Negative results with primers of single copy sequence BP283 (n = 22) is probably due to either very low amounts of DNA template in PCR master mix (C_t_ values ≤ 32 by the IS*481* assay) or presence of another species of *Bordetella* (31). This demonstrated that it was necessary to use more than one PCR target to ensure diagnosis of *B. pertussis* strains.

Statistical analysis of data showed that some factors have a significant effect on the rate of pertussis obtained by real-time PCR (P < 0.05). As shown in Table 3, treatment initiation is not considered as important inhibitors for the diagnosis of pertussis (P > 0.05). It may be due to some factors such as insufficient antibiotic treatment, specimen collection just after starting treatment or antibiotics resistance of the studied isolates.

**Table 3:** Comparison of positive results obtained from culture and real-time PCR assay using BP283 target.

Variables		No. (% in total specimens)	PCR positive (BP283)	Culture positive
age	≤2 years	510 (65.5)	79	9
	2 <age ≤ 10 years	147 (18.8)	10	1
	>10 years	84 (10.7)	6	1
antibiotic treatment	yes	583 (74.8)	74	7
	no	113 (14.5)	17	3
Gender	Female	364 (46.7)	35	4
	Male	415 (53.3)	65	7
Vaccination	yes	568 (72.9)	63	7
	no	138 (17.7)	27	3

In our study, the majority of PCR positive results and culture positive specimens were collected from young patients. Previous studies have shown that real-time PCR can be used to diagnose pertussis especially in infants less than 6 month of age (15). Although *B. pertussis* causes pertussis in adults and adolescent in some countries (2, 3, 41), this illness has still been seen significantly among children younger than 2 years of age with uncompleted vaccination in Iran (P < 0.05). The results obtained by Nakamura *et al* clearly indicate that adults had very low *B. pertussis* DNA loads in their nasopharyngeal swabs compared to infants and children. Antibiotic treatment and vaccination in adults support this hypothesis (42).

According to our results, vaccination has a significant effect on the rate of pertussis obtained from real-time PCR assay (P < 0.05). Our data showed that patients with no vaccination (19.5%) are more exposed to illness than patients who were immunized (11%). There was also a significant relationship between the results of the real-time PCR assay and gender of patients, males being more likely to be *B. pertussis* positive in our study (P < 0.05). In contrast, others have reported an opposite results (15).

In spite of importance of pertussis, lack of access to diagnostic methods, misdiagnosis, under reporting and differences in case definition between countries may result in false incidence of pertussis in both developed and less developed countries. The true number probably is higher than the number is reported from these countries (43, 44).

Based on pertussis incidence data from Center of Disease Control (CDC) in Iran, the incidence rate of pertussis was 1.2 cases per 100,000 populations and 1 case per 100,000 populations in 2008 and 2009, respectively (unpublished data form CDC of Iran). It seems that lack of enough awareness of correct sampling and accurate specimen transport to the laboratory is the main reason for great difference between the positive results obtained from two methods of detection (culture and real-time PCR) in our research. In addition, laboratory diagnosis methods such as culture and PCR are limited to certain laboratories in Iran.

Improvement in surveillance and disease control programs and increase in clinician awareness and reporting practices can be useful to estimate more accurate rates of pertussis in our country.
